# Nominalization and Alternations in Biomedical Language

**DOI:** 10.1371/journal.pone.0003158

**Published:** 2008-09-09

**Authors:** K. Bretonnel Cohen, Martha Palmer, Lawrence Hunter

**Affiliations:** 1 Center for Computational Pharmacology, University of Colorado School of Medicine, Aurora, Colorado, United States of America; 2 Department of Linguistics, University of Colorado at Boulder, Boulder, Colorado, United States of America; Northeastern University, United States of America

## Abstract

**Background:**

This paper presents data on alternations in the argument structure of common domain-specific verbs and their associated verbal nominalizations in the PennBioIE corpus. *Alternation* is the term in theoretical linguistics for variations in the surface syntactic form of verbs, e.g. the different forms of *stimulate* in *FSH stimulates follicular development* and *follicular development is stimulated by FSH*. The data is used to assess the implications of alternations for biomedical text mining systems and to test the fit of the sublanguage model to biomedical texts.

**Methodology/Principal Findings:**

We examined 1,872 tokens of the ten most common domain-specific verbs or their zero-related nouns in the PennBioIE corpus and labelled them for the presence or absence of three alternations. We then annotated the arguments of 746 tokens of the nominalizations related to these verbs and counted alternations related to the presence or absence of arguments and to the syntactic position of non-absent arguments. We found that alternations are quite common both for verbs and for nominalizations. We also found a previously undescribed alternation involving an adjectival present participle.

**Conclusions/Significance:**

We found that even in this semantically restricted domain, alternations are quite common, and alternations involving nominalizations are exceptionally diverse. Nonetheless, the sublanguage model applies to biomedical language. We also report on a previously undescribed alternation involving an adjectival present participle.

## Introduction

This work is a step toward understanding the syntactic and semantic aspects of verb meaning in the biomedical domain. The goal is to lay the groundwork for a set of representations of domain-specific verbs that is broad enough in its coverage to scale up to realistic problems in information extraction, and deep enough in its representation to support accurate extraction of information in the face of syntactic variability and to allow for the resolution of coreferential and related (e.g. elliptical) references in text. In an initial step, we sought to answer a very basic question: do alternations occur in biomedical texts? (*Alternation* is the term in theoretical linguistics for variations in the surface syntactic form of verbs.) We approached the problem by determining what the most frequent verbs are in biomedical text, then analyzing those verbs and their nominalizations in terms of the alternations that they participate in. Of the many classes of alternations that verbs participate in, we looked specifically at the passive alternation (Levin classes 5.1 *Verbal Passive*, 5.3 *Adjectival Passive*, and 5.4 *Adjectival Perfect Participle*) and at alternations related to transitivity (Levin class 1 *Transitivity alternations* and its descendants). We also report a previously undescribed alternation, *Adjectival Present Participle*. For the nouns, we examined alternations in the presence or absence of arguments and in the syntactic position of non-absent arguments.

One characteristic of alternations is that they preserve the underlying semantics of an assertion even in the face of syntactic variability. For example, one commonly known alternation is the passive alternation. One claim of an alternations-based approach to explaining syntactic/semantic relations is that in

- *FSH stimulates follicular development* (PMID 12021046) and
- *follicular development is stimulated by FSH* (PMID 6615964)

… the underlying semantics of the sentences, i.e. that *FSH* is the stimulator and *follicular development* is the thing that is stimulated, is the same, even though in the first sentence *FSH* is the grammatical subject and *follicular development* is the grammatical object, while in the second sentence *follicular development* becomes the grammatical subject and there is no grammatical object, per se. Alternations have been a topic of interest in the theoretical linguistics literature because they are thought to shed light on what is known in linguistics as the *mapping problem:* how it is that underlying semantics are realized in the syntax of sentences. One assumption of the model is that verbs with shared semantics will participate in the same alternations.

Alternations are of relevance to language processing and text mining because of the contribution that they might make to the development of broad-coverage rule- and pattern-based systems for relation extraction: if verbs with similar semantics do participate in the same alternations, then it might be possible to take advantage of this by inheriting or otherwise reusing abstract rules in broad classes of verbs. For example, if it turns out to be the case that transitive verbs share the trait of being able to occur in the passive alternation, then system developers might be able to write just two rules for extracting relations from active and passive sentences and share those between all transitive verbs, rather than writing a separate active rule and a separate passive rule for each transitive verb in the lexicon.

Levin (1993) [1] identified fifty major classes of alternations. That work also identified 49 major semantic classes of verbs, grouped according to the alternations in which they do and do not participate. (There are also subclasses of the fifty major classes of alternations and of the 49 major classes of verbs.) To illustrate the relationship between the semantics of related verbs and their shared syntactic behaviors, consider what Levin termed *calibratable change-of-state verbs*. These verbs–such as “increase”–share the semantic characteristics of a state-change in the logical object of the verb, and the syntactic behavior that when they are intransitive, the grammatical subject of the verb is the undergoer of the change (i.e., is the logical object). Thus, in

- *the addition of hCG alone significantly increased lyase activity in these cells* (PMID 2788776)

…the verb *increase* is transitive and *lyase activity* is both the grammatical and the logical object of the verb, while in

- *thecal lysase activity increased as the follicle matured* (same PMID)

…the verb is intransitive, and *thecal lysase activity* is the grammatical subject, but the logical object, of the verb. In contrast, the verb *breathe* can also be both transitive (*breathe pure air*, PMID 9636216) and intransitive, but unlike the case of *increase*, when *breathe* is intransitive, it is the logical subject that is the grammatical subject. So, we see *Rats…breathe spontaneously* (PMID 15693962), but it would be surprising to see an assertion about air breathing spontaneously. Thus, *increase* is clustered with other calibratable change-of-state verbs, such as *climb*, *decline*, *decrease*, *diminish*, *drop*, *fall*, *fluctuate*, *gain*, *rise*, and *vary*, but *breathe* is not, clustering instead with *bleed*, *cough*, *cry*, *dribble*, *drool*, *sweat*, and *vomit* (known as the *Breathe* subclass of the major class *Verbs of bodily processes*), Levin 1993 [1]:217–218). For an extended explanation of the phenomenon and theoretical implications of alternations, the reader is referred to the introduction to Levin (1993) [1]. For the natural language processing/text mining implications of the phenomenon, see below, as well as the *Discussion* section of this paper. To briefly anticipate what is made clear in the literature review in that section, we point out now that very few biomedical text mining systems cope with alternations in any robust way, and none come close to capturing the range of alternations that is attested in biomedical texts, particularly in the case of alternations that involve nominalizations.

The null hypothesis is that there are no differences in the incidence of alternations between general English and biomedical text. With respect to general English, Palmer et al. found that “Alternations in the realization of semantic arguments…turn out to be common in practice as well as in theory” (2005 [2]:101). However, scientific writing in semantically restricted domains is a classic example of a sublanguage (e.g. Sager 1972 [3], Sager 1986 [45], Harris et al. 1989 [5]), and Friedman et al. (2002) [6] have identified molecular biology abstracts specifically as fitting the sublanguage model. Sublanguages are frequently said to be characterized by a limited range of syntactic and semantic phenomena (see e.g. Sager 1972 [3] and Lehrberger 1982 [7]), which suggests that we might, in fact, not observe such alternations in this data.

Understanding the characteristics of a sublanguage has practical importance in the construction of natural language processing systems. However, it is not without theoretical interest, as well. Levin points out that

“…the hypothesis that the syntactic behavior of a word is fully semantically determined is not uncontroversial…Nevertheless…there are studies that show that this hypothesis receives substantial support, particularly in restricted domains…

…its success within limited, well-defined domains…depends in part on the investigation of intricate and extensive patterns of syntactic behavior.”

Levin (1993 [1]:13,16)

(We sketch the remainder of the paper here. The first section introduces the general topic of alternations and touches on the relevance of alternations to the question of whether or not the sublanguage model applies to biomedical texts. Its subsections introduce the topic of biomedical text mining, disuss at length the implications of the work reported here for the design of biomedical text mining systems, and review prior work on the related areas of biomedical predicate argument structures and semantics, as well as the prevalence of nominalization in boimedical texts. Other subsections present in-depth discussion of alternations and of nominalization, review various perspectives on nominalization itself, and present a discussion of the previously largely neglected topic of alternations involving nominalizations. The introduction concludes with the delineation of the contrasts between the work reported here and NomBank, the flagship project on annotation of the argument structure of nominalizations in General English. The *Materials and Methods* section describes the methodology for a pilot project that we performed on alternations in biomedical verbs, and then describes a much more extensive experiment involving nominalizations. The *Results* section presents a detailed analysis of the results of both experiments. One subsection is an extensive discussion of the issues involved in quantifying data on alternatioms that involve nominalizations, and reading it will make the data on the results for alternations involving nominalization easier to follow. A lengthy set of tables gives granular data on the alternations observed for each of the nominalized predicates under investigation. The *Discussion* section lists the implications of our findings for annotation efforts, reviews related work not already covered in the Introduction, and discusses the findings in terms of alternations and semantic representations, as well as explaining the relationship between our data and the predictions of the sublanguage model.)

### BioNLP: Biomedical natural language processing

BioNLP, or the application of natural processing to biomedical texts, primarily for purposes of text mining, has been a burgeoning area of research both within the computational linguistics community and within bioinformatics and computational biology. Data in Verspoor et al. (2006) [8] shows astonishing growth in the number of publications in the field just in the genomics domain alone; a significant body of research exists in the clinical domain, as well. The field is quite diverse, with work in recent years ranging from lower-level linguistic processing issues such as part-of-speech tagging and syntactic parsing to issues of deep semantic representation and analysis. Two motivations are commonly cited for work in this field: the need for practical applications for working bioscientists, and a desire to explore the potential of literature-based discovery and hypothesis generation. Reviews of the field appear fairly regularly, primarily in the bioscience literature; recent ones include Zweigenbaum et al. (2007) [9] and Cohen and Hunter (2008) [10].

### Implications for the design of biomedical text mining systems

The data described in this paper have implications for the design of biomedical information extraction systems. Despite a broad consensus that molecular biology texts fit the sublanguage model, we will show that even in the restricted domain of the abstracts that we examined in this study, syntactic alternations—both in verbs and in nouns—with consequences for information extraction (IE) systems are quite common.

At a minimum, such systems will need to be able to handle verbal passivization and the appearance of verbal predicators in adjectival positions. The adjectival alternations may be especially knotty—only two biomedical information extraction systems that we are aware of handle them, and then only for a single verb each: *bind* in the case of the ARBITER system (Rindflesch et al. 2000) [11], and *phosphorylate* in the case of the RLIMS-P system (Rule-based Literature Mining System for Protein Phosphorylation, Hu et al. 2005 [12], Narayanaswamy et al. 2005 [13], Yuan et al. 2006 [14]).

It is also clear that biomedical information extraction systems have a considerable way to go in their handling of the extant alternations involving the argument structure of nominalizations. A small number of extant biomedical information extraction systems tackle extremely limited sets of nominalizations: surveying the literature on biomedical information extraction, it is apparent that only a small number of implemented systems tackle nominalizations, and those that do so generally attempt to handle only a very small number of them, and only a minuscule fraction of the patterns in which their arguments can appear. For example, Ono et al. (2001) [15] attempt to handle the nominalizations *interaction*, *association*, and *binding*, and the relational noun *complex* (e.g. *Poll and Pob3 may form a complex*). Pustejovsky et al. (2002) [16] handle the single verbal nominalization *inhibition* and the single argument nominalization *inhibitor*. The RLIMS-P system (op cit) handles the single verbal nominalization *phosphorylation*. (This system is also the only one that explicitly targets NP-external arguments of the nominalization.) Goertzel et al. (2006) [17] describes a system that contains a “nominalization recognition” component; it is not clear what processing, if any, the recognized nominalizations undergo, and the system is currently in an unevaluated, prototype stage of development. Schuman and Bergler (2006) [18] do not include an interpretive component, but they demonstrate the ability to produce accurate syntactic attachment for post-nominal prepositional phrases using a corpus-based approach, achieving 82% accuracy for this task.

The most ambitious system that we are aware of with respect to nominalizations is Genescene (Leroy and Chen 2002 [19], Leroy et al. 2003 [20], Leroy and Chen 2005 [21]). This system tackles all verbs and nominalizations in the input; the only distinction between nouns and verbs in their system is that assertions from verbs without logical subjects (e.g. *expressed* in *p53 was expressed in five cases*, PMID 14631373) are extracted by the system, while assertions from nominalizations without logical subjects (e.g. *expression* in *expression of survivin and p53 in 61 cases of NSCLC*, PMID 16224523) are filtered out by the system. Genescene is built around manually-tuned regular expressions that are anchored by three prepositions: *of*, *in*, and *by*; any verbs or nominalizations that relate two noun phrases in the vicinity of these prepositions are extracted by the system. Assertions in which the related noun phrases correspond to UMLS categories are then returned to the user.

Genescene's focus on only three prepositions is sensible and well-motivated from a system construction perspective, and possibly from a precision-centric perspective. However, it limits coverage and therefore the ability of the system to scale. Our preliminary investigations of nominalizations have revealed that some common nominalizations can have arguments that are marked by a large number of prepositions. For example, of the five arguments of the verb *increase*, three were observed with arguments consisting of prepositional phrases headed by more than three prepositions (see Table 1); of the two arguments that were observed with fewer than three prepositions, neither of the prepositions associated with those arguments were Genescene's *of*, *in*, or *by*. (Not surprisingly, the group's publications generally eschew evaluation of coverage.)

**Table 1:** A sample predicate for which the three prepositions *of*, *in*, and *by* are insufficient for capturing all arguments.

Argument		Associated prepositions
Arg0	Causer of increase	*after*, *by*, *during*, *in*, *of*
Arg1	Thing increasing	*in*, *for*, *of*, *with*
Arg2	Amount increased by	*by*, *in*, *of*, *up*, *with*
Arg3	Start point	*From*
Arg4	End point	*to*, *with*

Our representation of this predicate is the same as PropBank's.

The work that we report here is significant in another way for Genescene: the NP-external arguments of nominalizations that our work shows to be common in biomedical texts are unlikely to be recovered very frequently by the Genescene finite-state approach. The computational power of Genescene is limited to that of finite state automata, the lowest degree of computational power (Partee et al. 1994) [22]. Although the Genescene system does apply a more powerful component for handling coordination, all other aspects of processing, including that of negation, is handled by finite-state automata.

Our work reveals other limitations on the potential coverage of a system like Genescene. Prenominal arguments—e.g. *phenobarbital induction* (induction *by* phenobarbital), or *trkA expression* (expression *of* trkA)—which our work shows to be characteristic of a number of biomedical nominalizations, are not related to the nominalization by prepositions and hence are never recoverable by Genescene.

The other architectural choice—and the one that we believe is the most crucial contrast between the Genescene system as a whole and any system informed by predicate-argument representations of semantics, or what Fellbaum (1993) [23] has referred to as *structural representations*, independent of whether what is being handled is a verbal or a nominalization-expressed assertion—has to do with the nature of the extracted assertion. Genescene's power in comparison to most of the systems that we have discussed here comes from the fact that rather than restricting itself to a small set of predicates, it applies a very general mechanism to extract *all* relations from the text (or at least all that are capturable within the limits of finite state power). It then filters out non-biomedically-relevant assertions, but then returns what is in essence a set of strings. There is no attempt to capture the similarity between predicates like *increase* and *enhance*, and no attempt to capture the difference between predicate-specific meanings of prepositions like *with* in *interaction of melittin with troponin C* (PMID 3579303) versus *transfection of fibroblasts with activated c-myc I* (PMID 2453829). In the former case, *with* links two things that act reciprocally, while in the latter, *with* links the direct object and indirect object. These very different relation/preposition mappings are not properties of the prepositions, but of the predicates; Genescene's potentially high coverage of predicates comes at the heavy cost of a complete lack of insight into the *meanings* of those predicates.

The consequent limitations on the long-term inability of its extracted assertions to support inferences of any but the most trivial sort are profound. In contrast, we propose a system driven not by prepositions per se, but by a lexicon of verb semantics that relates strings in text to a knowledge structure by the interaction between verb semantics, alternations, and the prepositions and other linguistic artifacts of those alternations. Note that our purpose here is not to criticize the Genescene system, which in a number of ways is quite innovative, but rather to show how our corpus-driven approach to the theoretical issues of alternations and of fit to the sublanguage model has practical and immediate consequences for the design of biomedical text mining systems.

The lack of attention to nominalizations in most biologically oriented text mining systems is an important finding for two reasons. One is that it suggests an obvious route for scaling up the productivity of language processing systems: nominalizations sometimes outnumber verbal forms significantly, and handling them can yield a substantial increase in recall. The other is that as the attention of the language processing community has slowly turned towards nominalizations, it is becoming clear that they are significantly more difficult to process than are verbs. Inter-annotator agreement in NomBank has generally been lower than in PropBank, and the tiny body of work on automatic semantic role labeling for nominalizations (Pradhan et al. 2004 [24], Jiang and Ng 2006 [25]) has reported generally lower performance than comparable systems for verbs. The data reported here is consistent with the hypothesis that multiple factors contribute to the greater difficulty of nominalizations as opposed to verbs. Two related ones are the presence of NP-external arguments (Meyers et al. 2004c [26]) and greater opportunities for argument-dropping without attendant syntactic cues, e.g. Arg0 omission without the passive construction. It is relatively difficult even for humans to distinguish these from each other, as evidenced by the confusion matrices in our nominalization annotation work.

### Biomedical predicate semantics and argument structures

Although syntactic alternations in biomedical text have not previously been studied, there are some precedents in the biomedical domain for work on the larger question of biomedical verbal argument structures. Friedman, Kra, and Rzhetsky (2002) [6] describe the broad features of the syntactic and semantic structures in molecular biology literature. Wattarujeekrit et al. (2004) [27] apply a lexical sampling method (Palmer et al., op cit, [2] p. 85) to construct predicate-argument representations for a small number of biologically relevant verbs. They give a FrameNet-like set of illustrative examples of naturally occurring sentences that illustrate the predicate-argument structures that they propose, but do not investigate alternations in argument structure, per se; Kogan et al. (2005) [28] extend that work to medical literature. Shah et al. (2005) [29] use Wattarujeekrit's representations as the inspiration for an information extraction system that was used to build a database of genes with alternative transcripts. Chou et al. (2006) [30] labelled PAS on a small set of verbs related to human blood cell transcription factors, and Tsai et al. (2006) [31] used that data to train a domain-specific automatic semantic role labelling system.

This prior work has primarily been concerned with either evaluating the possibility of building lexical resources for biomedical verbs (Wattarujeekrit et al. [27], Kogan et al. [28], and Chou et al. [30]) or with using these lexical resources as part of information extraction or semantic role labelling systems (Shah et al. [29], Chou et al. [30], and Tsai et al. [31]). There have been no attempts to use this domain to investigate basic issues of syntactic/semantic relations. Furthermore, one limitation of all of this work (in the biomedical domain—Meyers et al. 2004a [32], b [33], c [26] address this issue in the general English area) has been the restriction of its limited annotation efforts to verbs. Friedman et al. (op cit) [6] point out that in general, molecular biology publications tend to contain an enormous amount of information that is embedded in complex nominalizations. This was also noted by Tateisi et al. (2004) [34] in their work on annotation of predicate-argument structure in the GENIA corpus; they found that “…analysis of verb phrases is not sufficient because reactions and relations are often expressed in nominal phrases.” In the work reported here, we annotate and note variations in the syntactic realizations of the arguments of the nominalizations of common biomedical verbs.

### Alternations involving verbs

We examined the incidence of two types of Levin alternations: the passive alternation, and alternations related to transitivity.

The verbal passive alternation is almost caricatural of academic scientific prose. Biber et al. report that 25% of all finite verbs in academic prose are passives, versus 15% of all finite verbs in newswire text, and 2% of all finite verbs in conversation (1999 [35]:476). So, we expected to find a high incidence of passive constructions.

Alternations related to transitivity are the dominant characteristics of Change Of State verbs. Biologists conceptualize many molecular events as “state-changing,” so there seemed to be a potential for transitivity alternations in a biomedical corpus. However, we suspected that in fact it would be the case that these verbs appear consistently in just a single form, probably transitive.

Additionally, we present data on the occurrence of adjectival alternations. These include Levin class 5.3 *Adjectival Passive*, and also an adjectival present participle alternation, first reported here, that can occur both transitively and intransitively.

### Alternations involving nominalizations

The Levin-style literature on alternations has paid little attention to nouns. Levin (1993) [1] included limited discussion of zero-related nominals (this term contrasts with *zero-derived* in that it makes no assumptions about the direction of the derivation), but eschewed treatment of derivationally related nouns.

We develop the paradigm further by extending our analysis to derivationally related nouns. Our descriptive perspective on nominalizations is drawn primarily from Biber et al. (1999) [35], Quirk et al. (1985) [36], and Bauer and Huddleston (2002) [37]. The theoretical perspective comes from various publications by Meyers and his collaborators (Meyers, undated [38]; Meyers et al. 2004(a,b,c) [32], [33], [26]); in particular, the model of nouns as argument-taking predicators is consistent with Meyers's work (and with that of the PUNDIT project (Dahl et al. 1987) [39], of Johnston et al. 1995 [40], of Johnston and Busa 1996 [41], and of the FrameNet project, as well).

#### Argument nominalization versus verbal nominalization

For intelligibility, we give a short overview of typologies and terminology related to nominalizations.

Typologies of nominalization typically divide nominalizations into three broad categories. Morphologically unmarked nominalizations such as *increase* are known variously as *zero-related* (Levin) [1], *zero-derived*, or *converted* (Quirk et al. 1985 [36], Biber et al. 1999 [35], Bauer and Huddleston 2002 [37]).

Derivationally marked nominalizations are typically divided into two major categories.

Bauer and Huddleston contrast the broad categories of *person/instrument nominalizations* and *action/process/state nominalizations*. Their analysis focusses more on the behavior of the derivational morphemes involved than on any characteristics of the bases to which they are attached (beyond relatively superficial ones—specifically, part of speech). They lump person and instrument nominalizations together based on the observation that “…some processes…are used for both. Suffixation by *-er* is a clear example: compare *bottle-washer* (person) and *dish-washer* (instrument)” (p. 1697).

Meyers (2004a) [32] contrasts *argument nominalizations* and *verbal nominalizations*. His analysis focusses more on the nominalized entities than on the morphemes that are used to derive them. His *argument nominalizations* are nouns that denote a participant in some predicate—for example, *activator*. His *verbal nominalizations* are nouns that denote the predicate itself—for example, *activation*. Meyers's argument nominalization category does not posit any distinction between persons and instruments, which seems appropriate for this domain (see footnote 3, below).

Quirk et al. primarily organize nominalizations around the output of derivation, e.g. processes that produce concrete count nouns versus aggregate nouns versus abstract nouns. They describe two different -*ations* (p. 1551), and break down deverbal conversion into seven distinct categories.

Biber et al. (op cit) [35] are somewhat unusual in that they do not make a distinction beyond zero-derived or conversion nominalizations on the one hand, and suffixally derived nominalizations on the other (pp. 318–325).

Gerunds or participles are generally left out of these discussions, and we did not annotate them as nominalizations.

Of these various terms, we use *zero-related*, per Levin, and *verbal* and *argument nominalization*, per Meyers et al. We did not work with argument nominalizations at all in this project, so when we use the term *nominalization* without further qualification, we are referring to zero-related and verbal nominalizations.

### Nouns do participate in alternations

Although the classic Levin approach to alternations mostly eschews discussion of alternations involving nouns, there is ample evidence that nouns do participate in alternations. We show here that both transitivity and passivization alternations occur with English nominalizations.

#### Nominalization and passivization

Although not all uses of the term “passivization” are truly referring to passivity when applied to nouns, there does seem to be some consensus that passivization is a concept that is applicable to nouns. (Jespersen used the term *passive noun* to refer to “nouns designating the receiver of the action of a verb, words like *appointee*, *draftee*, *grantee*.” This is not the sense of passivity that we are concerned with here.) Work in theoretical linguistics has a long history of discussion of passivization and transitivity issues in relation to nominalization, reaching back at least to the transformational perspective of Lees (1963) [42] and extending through work from a cross-linguistic and typological perspective (Koptjevskaja-Tamm 1993) [43]. Recent work in theoretical linguistics has had a rich notion of passivity that clearly applies to nominalizations, differentiating between at least two kinds, viz. the *passive of a nominalization*, e.g. *…the receptor's phosphorylation by the kinase* (PMID 6090944) and “the nominalization *(–ity)* of a passive (*-able*)” (Roeper and van Hout 2006) [44], e.g. …*phosphorylability by cAMP-dependent protein kinase…* (PMID 9660676). (The distinctions between these types of passive nominalizations are based on a variety of forms of syntactic evidence, in addition to the obvious morphological differences. We have replaced Roeper and van Hout's examples with comparable ones from the biomedical literature.) Roeper and van Hout's passive nominalizations are explicitly contrasted with active nominalizations, e.g. *RK's phosphorylation of R* (PMID 10448166).

#### Nominalization and transitivity

It is also clear that notions of transitivity can be applied to nominalizations. For example, Koptjevskaya-Tamm refers to transitive and intransitive nominalizations and presents a small typology of their argument types (1993 [43]:11–12). Similarly, Quirk et al. discuss *-tion*-derived nominalizations with respect to transitivity.

#### The field of alternations for nominalizations is much larger than this

However, there is an enormous amount to be observed about alternations in the argument structure of nominalizations that transcends the small number of labels (active vs. passive, transitive vs. intransitive, etc.) that we have applied to verbs in this work. In particular, there are differences in the presence or absence of arguments, and for arguments that are not absent, there are differences in the position of arguments, which may be either within or external to the noun phrase (NP); for arguments that are NP-internal, there are differences in whether the argument appears to the left or to the right of the nominalization. For example, the Arg1 of a “transitive” nominalization can precede it as a bare noun, e.g. *TRKA expression* (5 tokens in the PennBioIE corpus—*TRKA* is the name of a gene) or can follow it within a prepositional phrase, e.g. *expression of TRKA* (one token in the PennBioIE corpus). If we consider three things: (a) that any argument of a nominalization may, in theory, be absent, be present but external to the NP, or be present within the noun phrase in either of two structurally distinct positions; and (b) that in general, perhaps *all* nominalization tends to be similar to verbal passives in permitting the omission of agents, and similar to verbal intransitives in permitting the omission of patients, and (c) that there are more than just the two arguments Arg0 and Arg1 to take into account in considering nominalizations, then it perhaps makes less sense to focus on squeezing the potentially enormous number of distinct patterns of argument realization for a given nominalization into the same categories as we apply to verbs than it does to try to characterize the range of possible alternations that is attested.

For these reasons, we refer to any combination of the set of values for each ArgN of a nominalization, drawn from the set of four values listed and described in Section *Representation of arguments*, as “an alternation,” without attempting to name them further. We will consider a set of data like the following to attest five distinct alternations:

- ***activation*** *[of molecular oxygen_Arg1_] [by alkaline hemin_Arg0_]*
  alternation: [Arg0 post-nominal] [Arg1 post-nominal]
- *[K(ATP)_Arg1_]* ***activation*** *[by cromakalim_Arg0_]*
  alternation: [Arg0 post-nominal] [Arg1 pre-nominal]
- *[mutational_Arg0_]* ***activation*** *[of the ras genes_Arg1_]*
  alternation: [Arg0 pre-nominal] [Arg1 post-nominal]
- *[H-ras, K-ras, and N-ras oncogene_Arg1_] [mutational_Arg0_]* ***activation***
  alternation: [Arg0 pre-nominal] [Arg1 pre-nominal]
- ***activation*** *[of an N-ras oncogene_Arg1_]*
  alternation: [Arg0 absent] [Arg1 post-nominal]

…and to a set of data like the following as attesting three tokens of the same alternation:

- ***activation*** *[of ras proto-oncogenes_Arg1_]*
- ***activation*** *[of MMP-2_Arg1_]*
- ***activation*** *[of CYP1A1 gene transcription_Arg1_]*

…since they all have the pattern [Arg0 absent] [Arg1 post-nominal].

### Contrasts between this work and NomBank annotations

Our annotation of nominalizations attempts to be consistent with NomBank's guidelines to the greatest extent possible. Our approach and guidelines differ from NomBank's in three ways:

- Markables: The NomBank project does not mark nominalizations that have no argument instances (see p. 7 of Meyers (undated) [38], and pp. 1 and 2 of Meyers et al. (2004a) [32] to validate this and for their rationale). We do annotate these nouns, since they are cases of intransitives.
- Spans: NomBank arguments are mostly bounded by some syntactic element in the Penn Treebank annotation. We worked without syntactic annotation, which introduced issues of span selection (see Section*Span selection* below).
- NP-external arguments: In principle, NomBank constrains its use of NP-external arguments to specific syntactic constructs. Like NomBank, we ruled out arguments whose identification would rely entirely on inference, but unlike NomBank, we did not attempt to place any syntactic constraints on what could count as an NP-external argument (beyond the obvious requirement that it be external to the NP).

## Materials and Methods

### Materials

We used release 0.9 of the PennBioIE corpus (Kulick et al. 2004) [45] and release 3.0p of the GENIA corpus (Kim et al. 2003) [46]. (Due to space considerations, in all table captions we refer to the PennBioIE corpus as the BioIE corpus.) Both of these corpora are composed of the titles and abstracts of scientific journal articles. The PennBioIE corpus is divided into two parts reflecting two distinct semantic domains: CYP450 (*cytochrome P450* is the name of a family of proteins involved in, among other things, determining individual responses to pharmaceutical agents), and Oncology. Although the PennBioIE corpus is intended to have fully curated part-of-speech tags and syntactic parses, various issues with the current (early and pre-1.0-release) version made it impractical to make use of much of the annotations.

The limited data that we present from the GENIA corpus is primarily for comparison of word distributions in the two corpora—all data on alternations in the paper is from the PennBioIE corpus.

All examples in this paper are drawn from naturally occurring data. Almost all examples are drawn from the PennBioIE corpus. In the case of such examples, we do not give further citations. For the occasional examples that we draw from other published articles, we identify the source by giving its *PubMed identifier*, or PMID. PubMed is a freely available database of scientific articles made accessible by the National Library of Medicine.

### Finding the frequent domain-specific predicates

The first step in this project was to determine the most frequent domain-specific predicates represented in the data. We first extracted all verb tokens from both corpora by using egrep to search for tokens whose tags matched the pattern **VB.?** in the PennBioIE **.mrg** files and the GENIA **GENIAcorpus3.02.pos.txt** file. (This is a potential source of a small amount of noise in the PennBioIE data, since not all POS tags are curated in that data. Fifty tokens from the PennBioIE data, including numerals, punctuation marks, and single letters, were clearly mis-tagged as verbs.) We then collapsed inflected forms of verbs by applying the Porter stemming algorithm (Porter 1980) [47], using a publicly available implementation from the Tartarus web site. We filtered the lists of verbs from the combined halves of the PennBioIE corpus, the separate halves of the PennBioIE corpus, and the GENIA corpus by removing the most common non-domain-specific verbs (e.g. **be, use**, and **have**) found in the combined PennBioIE corpus. (Note that the boundary between “domain specific” and “general English” verbs is not always clear-cut. For example, scientific writing commonly personifies non-animated agents (Biber et al.,p. 372), and the molecular biology genre is not known to be an exception to this generalization, so e.g. *occur*, which might not seem like a biologically relevant verb, often encodes relations between biological processes, entities, and events. For example, in *The metabolism of saturated nitriles*, *including acetonitrile*, *has been assumed to occur by a cytochrome P-450-dependent oxidation at the alpha carbon…* the verb *occur* is what asserts the relationship between metabolism of nitriles and CYP450-dependent oxidation.) The result was a list of the domain-specific verbs in each corpus, ordered by frequency. In the analysis that follows, we concentrate on the ten most common domain-specific verbs.

Having determined the ten most frequent verbs, we then retrieved all sentences containing any form of these verbs from the CYP450 portion of the PennBioIE corpus. (This resulted in a data set with somewhat different verb distributions from the corpus as a whole, but a much more clearly defined semantic domain.) For each verb, we extracted all tokens of each of four word forms independently: the bare stem/non-third-person-present-tense form (e.g. *inhibit*, *induce*, and *increase*), the third person singular present tense form (e.g. *inhibits*, *induces*, *and increases*), the present participle (e.g. *inhibiting*, *inducing*, and *increasing*), and the past tense/past participial form (e.g. *inhibited*, *induced*, and *increased*).

There were a number of potential sources of noise in this process:

- We considered adjectival passives and perfect participles to be verbs, since they constitute alternations (Levin classes 5.3 and 5.4). However, they generally are tagged in PennBioIE not as verbs, but as adjectives. This causes a discrepancy between the verb counts that we came up with when determining the most frequent verbs and the count of tokens that we extracted when retrieving sentences containing verb forms. The latter is higher, since it contains tokens that were tagged as JJ (the Penn Treebank tag for an adjective).
- Our sentence segmentation was naive, and we occasionally retrieved fragments smaller than a sentence.
- After completing the verb analysis, we belatedly realized that our retrieval script was case-sensitive, so we missed some verb tokens. The number of such tokens was small—for example, the number of tokens of *associated* with and without case sensitivity is 108 and 109, of *treated* is 327 in both cases, of *activated* is 82 and 83, of *containing* is 144 in both cases, and of *expressed* is 175 and 180.

### Annotation of verbs

We made two passes through most of the data, eventually settling on a single set of nine tags that could be applied to all verbs and all of their forms. Table 2 gives the tag set, with examples. Eight of the tags capture the three axes of active versus passive, transitive versus intransitive, and verbal versus adjectival. A ninth tag was used only for nouns. We used X's to indicate when we could not determine a value. In practice, we almost always used it for passives for which we could not determine transitivity versus intransitivity. (The notion of a passive intransitive might seem counterintuitive, but see Levin (1993:87), and note that two of her examples (*collapsed lung* and *slipped disc*) are medical in nature.) Two of the tags were never used: *PI*, and *pi*. All of the terms correspond to some Levin alternation, with the exception of the two tags that we used for prenominal adjectival present participles, *at* and *ai*. We suggest that although they do not correspond to any alternation in Levin (1989), they are parallel constructions to the adjectival passives that manifest as past participles. Correspondence with Beth Levin did not yield a counterargument.

**Table 2:** The tag set for verbs.

Tag				Example
AT	active	Transitive	verbal	*Halofantrine and chloroquine * ***inhibit*** * CYP2D6 activity…*
AI	active	intransitive	verbal	*Thus, thecal lyase activity * ***increased*** * as the follicle matured…*
At	active	Transitive	adjectival	*Selenoxidation by flavin-* ***containing*** * monooxygenases…*
Ai	active	intransitive	adjectival	*In the presence of * ***increasing*** * concentrations of BH4…*
PT	passive	Transitive	verbal	*Diazinon is * ***activated*** * by CYP2C19 in human liver.*
PI	passive	intransitive	verbal	Not attested
Pt	passive	Transitive	adjectival	*Cortisol-* ***induced*** * aromatase activity in Om adipocytes…*
Pi	passive	intransitive	adjectival	Not attested
N	Noun			*This * ***increase*** * was partially inhibited by carbon monoxide…*

### Inter-annotator agreement for verb labeling

105 tokens were annotated by a second annotator. We calculated inter-annotator agreement (IAA, a quantitative assessment of the extent to which multiple annotators' judgements agree with each other, which is generally thought to give a direct measure of the reliability of annotations and thereby an indirect measure of the validity of annotations (Artstein and Poesio 2007) [48]) as the percentage of tags on which we agreed divided by the total number of tags. The second annotator was first provided with written directions. We then reviewed a small set of examples, and the second annotator labelled a few examples until they felt comfortable.

The 105 tokens represent a stratified sample distributed equally across the seven attested tag types—fifteen of each, for a total of 105. Within the tag types, we biased our selection of target words towards the most ambiguous ones. For example, *activated* appears in active transitive verbal (AT), passive transitive verbal (PT), and passive transitive adjectival (pt) forms, so we included more samples of it in the test set than of *occurred*, which only appeared in active intransitive verbal form. They were presented to the second annotator as we annotated them—grouped by word form (e.g. all tokens of *increase* together, all tokens of *increases* together, etc.); within a word group, ordered by UNIX sort of the source filename.

### Methods: nominalizations

#### Representations of nominalizations

The annotation work for the verb portion of this project was approachable based on obvious and implicit assumptions about the argument structure of the verb types and their tokens in the corpus. In contrast, the annotation work for the nominalization portion of this project required explicit definitions of predicate-argument structure as a first step in the analysis (see Meyers, who used the PropBank representations whenever possible). As work by Wattarujeekrit et al. [27], by Kogan et al.[28], and by our group (Cohen and Hunter, 2006) [49] has pointed out, the overlap between biomedical-domain-specific verbs and verb senses and the publicly available resources is not high. (For a dissenting opinion, see Tsai et al. (2006) [31], but their view is not widely held, and the data in their own paper actually argues in favor of the majority opinion on this.) To assemble our PAS, we consulted four sources, using their representations where they were applicable, and adding to or modifying them when they were not. The full set of ten PAS that we settled on is available as a Protégé project. Three of the sources are publicly available; the fourth, reported in Tsai et al. (2006) [31], is not, but the authors were kind enough to share it with us. The four resources that we consulted were:

- PropBank (Palmer et al. 2005) [2]: this is a set of about 6500 PAS that has been shown to suffice for representing WSJ data.
- BioProp (Tsai et al. 2006) [31]: this is a set of PAS for 30 biomedical-domain-specific verbs. It is built on the assumption that PropBank PAS should be used whenever they exist, and should be augmented only when a biomedical verb is completely absent from PropBank.
- PASBio (Wattarujeekrit et al. 2004) [27]: this is a set of 31 predicates (distributed across 29 verbs) in the biomedical domain. It maps to PropBank PAS and WordNet senses to the greatest extent possible, but adjusts PropBank representations to reflect biomedical senses; they found this to be necessary quite often, with only six of the 29 verbs that they examined having the same sense and same argument structure as the corresponding PropBank verbs.
- FrameNet (Fillmore et al. 2003) [50]: this is not a set of PAS per se, but its frames are mapped to lexical units, and when the verbs under analysis in this project were represented there, we considered the FrameNet core arguments for representational suitability for the biomedical senses of those verbs.

**Figure 1:**
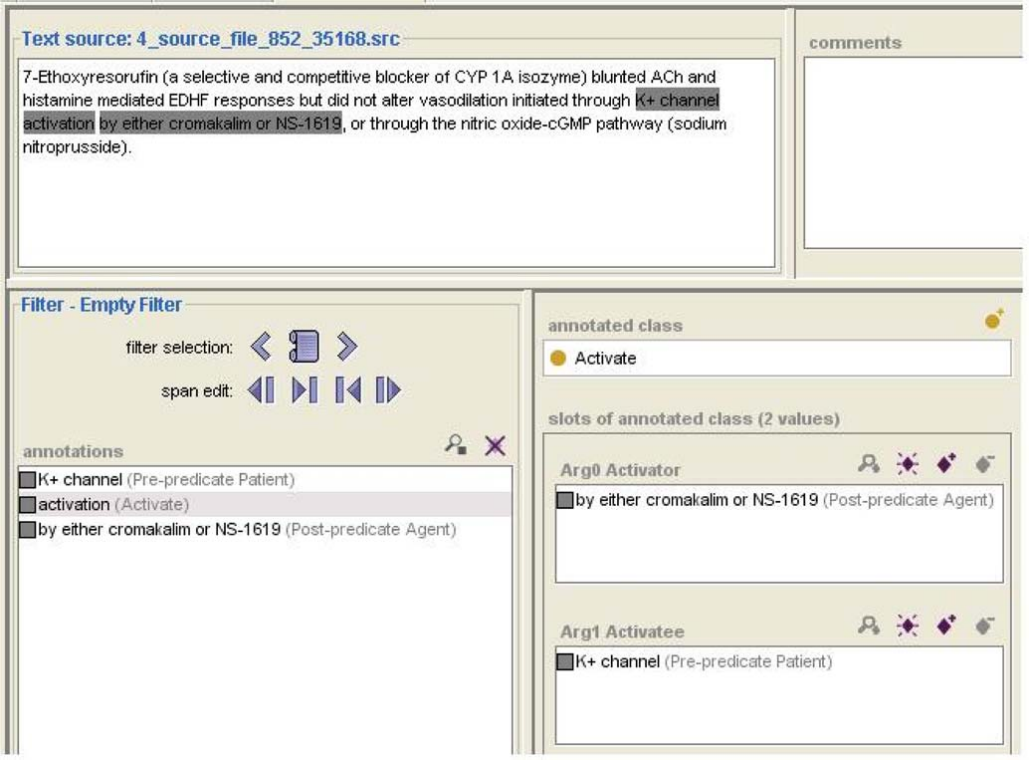
A screen shot showing the representation of a predicate and the annotation of a token of that predicate in text. The top pane shows the textual data. The slots in the bottom right pane indicate the arguments of the predicate *activate:* an Arg0, the *activator*, and an Arg1, the *activatee*. The subpanes corresponding to those slots show the text in which the arguments are instantiated—*by either cromakalim or NS-1619* and *K+ channel*—and indicates the syntactic position—post-predicate and pre-predicate, respectively—of each. The bottom left pane lists all segments of text that have been annotated. Since the predicate itself is highlighted in the bottom left pane, its argument structure and arguments are displayed in the bottom right pane.

Our representations for nominalizations consisted of framesets, or groupings of a wordform; a sense label, where differentiation was needed; and a set of argument slots. Figure 1 shows the argument slots for *activate*—Arg0 is the activator, and Arg1 is the activatee. Except for the obvious case of negatives, we did not assume any distinction between core and adjunct arguments (see Cohen and Hunter (2006) [49] for a review of the controversy over this distinction in the biomedical domain). Like PropBank, we reserved the Arg0 slot for agents. Arity of the argument sets ranged from a low of one (for *occur*) to a high of five (for *increase*).

#### Representation of arguments

We labelled arguments in text with one of five categories, primarily differentiated from each other on positional criteria:

**Pre-nominal**: Within the NP, as a prenominal modifier.
**Post-nominal**: Within the NP, as a post-nominal modifier.
**No argument present**: Completely absent.
**NP-external**: Argument is external to the NP of the noun to which it is an argument. See below for a fuller discussion of NP-external arguments.
**Can't tell**: Could not decide whether the argument is present or not, or if so, which NP it maps to. See below for a fuller explanation.

**Pre-nominal**: Pre-nominal arguments occur as leftward modifiers of the nominalization, within the noun phrase, e.g. *phenobarbital induction* and *trkA expression*. They are most often (in this data, and probably elsewhere) nouns. However, they very occasionally also occur as adjectives. We followed the NomBank guidelines (Meyers, undated) [38] for those cases, annotating adjectives only when they had argument-filling roles, but not otherwise. Thus:

- *Mutational activation of the beta-catenin proto-oncogene*: A mutation is doing the activation, i.e., is the Arg0 of *activation*, so *mutational* is annotated.
- *transcriptional activation of AP-1*: *transcription* is neither what is being activated (i.e., is not the Arg1 of *activation*) and is not what is doing the activation (i.e., is not the Arg0 of *activation*), so it is not annotated. (Transcriptional activation is a kind of activation.)
- *Surgical treatment of anterior callosal tumors*: Surgery is the “instrument,” i.e. is the Arg2 of *treatment*, so *surgical* is annotated.
- *metabolic activation of DMBA*: Metabolism is neither what is being activated (i.e., is not the Arg1 of *activation*) nor is what is doing the activation (i.e., is not the Arg0 of *activation*), so it is not annotated.

This is discussed in greater detail, and with more examples, in our annotation guidelines.

**Post-nominal**: Post-nominal arguments occur in prepositional phrases to the right of the nominalization, within the noun phrase. For example, ***increases*** *[of oxygen_Arg1_] [from 10_Arg3_] [to 70%_Arg4_]* has three post-nominal arguments—the Arg0 (thing increasing), Arg3 (starting point of the increase), and Arg4 (end point of the increase). (If our analysis of the noun phrase seems questionable, the full sentence is *Post hoc analysis of the data showed that*, *with the greatest concentration of propofol (1000 microM)*, *there was increasing inhibition of metabolism of midazolam with increases of oxygen from 10 to 70%*.)

**No argument present**: Arguments with no surface realizations were explicitly marked as absent. (This had the advantage of allowing for a quality check on our annotations with the Knowtator tool, since it allowed us to distinguish between an argument slot that was empty because we had not dealt with it yet versus an argument slot that actually had no argument in the data.) For example, for the predicator *Induction* in

- *Induction followed a slower kinetic compared to that observed for c-fos and NGF1A*

…none of the three arguments of *induce* are present: neither the Arg0 Inducer, nor the Arg1 Induced, nor the Arg3 Extent of Induction.

The explicit marking of these absent arguments is a deviation from the NomBank guidelines, but is similar to “null instantiations” in FrameNet.

**NP-external arguments** were the most troublesome category in the annotation scheme. Meyers et al. (2004c) [26] is an extended treatment of the notion that nominalizations can have arguments that occur outside of the noun phrase of the nominalization. For example, in

- *[this enzyme_Arg1_] can undergo activation at Ph 5.5* (PMID 1482371),

…*this enzyme* is an NP-external argument of *activation* (see Meyers (undated) [38], p. 64, for the status of *undergo* as a support verb). They present reasoned arguments for why specific syntactic constructs should be analyzed in this way, and a number of similar issues are discussed in Fillmore et al. (2003) [50]. The main difference between Meyers et al. and Fillmore et al. with respect to this is that Meyers et al. extend the licensors of NP-external arguments beyond the support verbs and transparent nouns of FrameNet to a wider variety of multi-word expressions. The argument is reasonable; from an annotation perspective, the challenge is to draw the line between arguments that are actually licensed by some syntactic criterion, and ones that are only licensed by inferential processes or by zero anaphoric reference (see Dahl et al. 1987 [39] for a system that implements this distinction). Like NomBank, we ruled out arguments whose identification would rely entirely on inference, but unlike NomBank, we did not attempt to place any syntactic constraints (in terms of SUPPORT constructions, transparent nouns, etc.) on what could count as an NP-external argument (beyond the obvious requirement that it be external to the NP). Looking at the examples in Meyers (undated) [38], it is not clear to me that NomBank necessarily respects them, either; not surprisingly, this category is a large contributor to annotator disagreement in the NomBank project (Meyers et al. 2004b [33]:28).

**“Can't tell”**: The classification scheme included a label for the specific case where an annotator could not tell whether or not an argument was present. The “can't-tell” class was used for two distinct situations: where the annotator could not tell because the data was defective (almost always due to a fault in the sentence segmenter of our search script), and where the annotator could not tell because the example was genuinely ambiguous or otherwise unclear. In future work, it would be useful to differentiate between these cases—the former are artifacts of the text processing strategy, while the incidence of the latter is a genuine index of the difficulty of the task.

#### Span selection

Annotation projects similar to this one have typically been carried out using text sources that already had hand-curated syntactic parses, reducing the question of what span of text to annotate for a given argument to the question of node selection (for the distinction between node selection or *role identification* and *role classification*, see Palmer et al. 2005 [2]). Due to technical issues with the pre-1.0 version of the PennBioIE corpus that we used as our source corpus, we had no recourse to this, and the annotation process therefore involved making decisions about the text span to select, in addition to the class assignments (i.e. role/argument assignment, as well as the “syntactic” classes of pre-nominal, NP-external, absent, etc.). Even in the case of projects involving pre-parsed data, related issues arise, such as the PropBank issue of whether numbered arguments in prepositional phrases should be labelled at the level of the NP or at the level of the parent PP (Palmer et al. 2005 [2]); see also Meyers (undated) [38]. In our case, part of the motivation for gathering data on these alternations related to nominalizations is to use the data to write patterns for language processing systems, so information about the identity of prepositions is crucial. In brief, our span selection guidelines were:

- When arguments are post-nominal, include the preposition (and the rest of the noun phrase, including all material to the left of the noun). For example, in *activation [of the beta catenin gene]*, *of the* is included in the span.
- When arguments are pre-nominal, include leftward material up to the determiners, but do not include prepositions. For example, in *for [quinidine] inhibition*, *quinidine* is marked as the Arg0 of *inhibition*, but *for* is not included in the span.
- When arguments are NP-external, include content words only. For example, in ***activation*** *undergone by [transducin]*, the *by* that precedes *transducin* is not included in the span.

Much more explicit guidelines for span selection are included in the annotation guidelines, along with numerous examples.

#### Annotation process

We did annotation of nominalization arguments using the Knowtator text annotation tool (Ogren 2006a,b [51], [52]). When a nominalization is identified and labelled in a text source, Knowtator displays a set of argument slots for that nominalization (See Figure 1). It provides a very simple interface for mapping the realizations of those arguments to the nominalization itself, and for labelling them or for indicating them as absent (or “can't tell”) when appropriate. For example, in the figure, the Arg0 is the text string *by either cromalakin or NS-1619*, which has been labelled as [post-predicate]. The Arg1 is the text string *K+ channel*, which has been labelled as [pre-predicate]. (Knowtator also has functionality for managing annotator IDs, merging the work of multiple annotators, and automatically calculating an extensive set of inter-annotator agreement metrics.)

We first annotated all 746 tokens. A second annotator then annotated a stratified sample of 114 (15%) of these.

#### Nominalization annotation data selection

We attempted to annotate 100 tokens of each verbal nominalization—50 from each section of the corpus. Table 3 shows the number of tokens that we actually annotated for each nominalization. If one section had fewer than 50 tokens of a given nominalization, we annotated extra tokens from the other section to make up the difference. Some nominalizations had only a very small number of tokens in either corpus, so the total number of nominalizations that we annotated was only 746.

**Table 3:** Counts of annotated tokens.

Nominalization	BioIE (both)	BioIE-P450	BioIE-Onc
*inhibition*	100	50	50
*induction*	100	50	50
*increase*	100	50	50
*expression*	101	50	51
*association*	91	14	77
*mediation*	2	1	1
*containment*	1	0	1
*occurrence*	51	3	48
*treatment*	96	46	50
*activation*	100	50	50

Rows are ordered by frequency of the corresponding verb in the BioIE corpus. The goal was 100 tokens per type.

We retrieved sentences containing the specific lexical items from the PennBioIE corpus using the script described above. In some cases, the naive sentence segmentation of the script returned defective sentences. The search returned both singular and plural nominalizations; we did not attempt to balance them, except in the case of *increase(s)*, which required special handling since its zero derivation makes it indistinguishable from a verb without access to POS tagging. For the case of *increase(s)*, we used the data that we had labelled in the first part of this study—the tag set that we used there included N(oun), so we were able to retrieve hundreds of samples of both the singular and the plural form easily.

#### Inter-annotator agreement for nominalizations

15% of the data (114 nominalization tokens) were annotated by a second annotator. The 114 tokens represent a stratified sample distributed across the eight nominalizations that had enough tokens in the data to allow us to train the second annotator with data that we would not be using to calculate IAA. (This meant excluding the two nominalizations *mediation* and *containment*, which only had two and one tokens each in the corpus, and including all of the other eight.) They were presented to the second annotator as we annotated them—grouped by nominalization (i.e. all tokens of *inhibition* together, all tokens of *induction* together, etc.), ordered by the order in which they were retrieved from the corpus.

The preferred metric to report for inter-annotator agreement is the Kappa statistic. We calculated inter-annotator agreement as F-measure, treating one annotator as the gold standard. (The calculation is not affected by which annotator is treated as the gold standard.) When the probability of chance agreement *P(E)* approaches zero, this is equivalent to Kappa (Hripcsak and Rothschild 2005 [53]); since the data is not parsed (and therefore there are no constraints imposed on span selection) and the number of potential slot-filling classes is large and includes “absent argument” and “can't tell” options, the P(E) arguably *is* essentially zero.

Although we collected data for all of the nominalizations (and for intra-annotator agreement, as well as for inter-annotator agreement), software problems with the Knowtator tool necessitated painful manual collection of the IAA data and calculating IAA manually, making determination of IAA practical for only a subset of the verbs and slots. To get the most stringent evaluation possible under the circumstances, we calculated inter-annotator agreement only for the two nominalizations for which we expected the lowest agreement. Impressionistically, we found *activation* to be the most difficult nominalization to annotate (almost all of the consultations with “native speakers” that we mention in the Acknowledgments were about this nominalization), and *expression* is a notoriously difficult concept to represent in this domain (see Cohen and Hunter (2006) [49] for a discussion of the issues), so we chose those two verbs. Additionally, inter-annotator agreement will presumably be lower than intra-annotator agreement, so the limited IAA data that we present for nominalizations in Section *Inter-annotator agreement for nominalizations* should represent the lower bound on both inter-annotator and intra-annotator agreement. We calculated confusion matrices for the various labels and subparts of the task.

## Results

### The most frequent domain-specific verbs

Table 4 lists the most common domain-specific verbs (i.e. after filtering out non-domain-specific ones) for the PennBioIE corpus as a whole and for its two divisions. GENIA data is given for comparison.

**Table 4:** Most common domain-specific verb lemmas.

BioIE (both)	BioIE-P450	BioIE-Onc	GENIA
inhibit (637)	inhibit (615)	associate (101)	induce (1322)
induce (310)	induce (238)	identify (84)	activate (1122)
increase (257)	increase (188)	occur (81)	express (827)
express (135)	treat (102)	activate (78)	inhibit (811)
associate (133)	decrease (102)	include (73)	demonstrate (734)
mediate (130)	catalyze (100)	induce (72)	bind (730)
contain (125)	mediate (94)	contain (70)	increase (700)
occur (124)	reduce (74)	increase (69)	regulate (659)
treat (118)	follow (69)	express (68)	contain (595)
activate (116)	stimulate (68)	analyze (62)	require (555)

### Alternations: verbs

Passive and transitive alternations occurred frequently. Table 5 gives a broad overview of what alternations occurred with which verb forms. Incidence of the individual alternations is given in the text that follows.

**Table 5:** Alternations involving BioIE top-10 verbs in the CYP450 section.

Lemma	bare	-s	-ing	-ed
inhibit	AT, AI	AT	AT, at, ai	AT, AX, PT, pt
induce	AT	AT	AT, at, ai	AT, PT, pt
increase	AT, AI, N	AT, AI, N	AT, AI, ai, X	AT, AI, ai, PT, X, PX, px
express	AT	AT	AT, at	AT, PT, pt, PX
associate	—	—	—	PT, pt
mediate	AT	AT	AT	PT, pt
contain	AT	AT	AT, at	AT, PT
occur	AI	AI	AI, ai	AI
treat	AT	—	AT	PT, pt
activate	AT	AT	AT, at	AT, PT, pt

Dashes indicate that a verbal form did not occur in the corpus. *AT* = active, transitive, verbal. *at* = active, transitive, participial modifer. *N* = nominalization. *P* = verbal passive. *p* = adjectival passive. *PT* = verbal passive, transitive. *pt* = adjectival passive, transitive.

### Passive alternations

As Table 6 shows, alternations related to passivity were quite frequent in this data. 473 of the verb tokens were passive, while 1,142 were active, so passives constituted 29.3% (473/(473+1,142)) of all verb tokens. Two types of passive alternations—the verbal passive (Levin class 5.1), and the adjectival passive (Levin class 5.3)—were well represented. Verbal passives occurred in two very different syntactic structures—as main-clause verbs, e.g.

**Table 6:** Passive alternations in the 10 most common verbs.

Alternation	count
Verbal passive (5.1)	287
Adjectival passive (5.3)	186
Adjectival perfect participle (5.4)	0
All passive (5.1+5.3+5.4)	473
All active	1,142

The top half of the table breaks down the passives by type. The bottom half gives the sum of the passives, and the corresponding number of actives.

- *This protection* ***was associated*** *with decreased formation of the toxic metabolite*…),

…and as the matrix verb of a post-nominal relative (typically a reduced relative) clause, e.g.

- *mortality* ***associated*** *with the development of opportunistic infections*).

### Alternations related to transitivity

Table 5 gives the argument structures in which the various verbs appeared, and Table 7 gives the incidence of transitive and intransitive verb tokens for the verbs that appeared with more than one transitivity-related valence structure. Seven of the ten most common verbs appeared only as transitives. The three most common verbs appeared predominantly as transitives. All three showed at least one example each of an intransitive, but only the one clearly change-of-state verb, **increase**, showed a large proportion of intransitives (96 transitive, 60 intransitive). Only one verb, **occur**, always appeared intransitively.

**Table 7:** Incidence of transitives and intransitives for verbs that varied.

Lemma	Trans.	Intrans.	Couldn't tell
inhibit	539	2	1
induce	187	1	0
increase	96	60	5

### Adjectival alternations

Alternations related to adjectival forms of the verb were quite common. In the 10 most common verbs, there were 294 adjectival forms, versus 1,321 verbal forms, so 18.2% (294/(294+1,321)) of all non-noun tokens were involved in this alternation. Two different forms of adjectival alternation were well-represented, with both passive and active (present participial) forms occurring. Table 8 gives the distribution across the various alternations. Transitive and intransitive forms both appeared amongst the present participial forms; surprisingly, the incidence of the transitive form is higher.

**Table 8:** Adjectival alternations among the ten most common verbs.

Alternation	count
Adjectival passive (5.3)	184
Adjectival perfect participle (5.4)	0
Adjectival “X”	2
Present participial adjective (transitive)	59
Present participial adjective (intransitive)	49
Present participial adjective (all)	108
All adjectival	294
All non-adjectival verbs	1,321

The top half of the table gives the breakdown among adjectival types. The bottom half of the table gives the sums across all types.

### Alternations: nominalizations

#### Quantifying data on alternations for nominalizations

It is less obvious how to quantify the data on alternations in a way that will allow us to test the predictions of the sublanguage hypothesis for nominalizations than for verbs. Tokens of verbs are straightforwardly categorizable in terms of three (mostly orthogonal) binary categories—active vs. passive, transitive vs. intransitive, and verbal vs. adjectival. In contrast, in the case of nominalizations of those verbs, there are multiple arguments to be considered in relation to each other, and each argument may vary in terms of presence or absence, and in the case of present arguments, may vary with respect to location inside or outside of the noun phrase, and in the case of arguments within the noun phrase, may vary with respect to position relative to the nominalization. (There is an additional possible axis of variability with respect to whether the nominalization is the head of the noun phrase or is itself a pronominal modifier, e.g. *inhibition* in *inhibition constant*. We annotated such nominalizations when they occurred, but did not consider them separately in the analysis. Impressionistically, our data seem to support Meyers et al.'s claim that non-head nouns have the same argument structures as head nouns, but we do not have enough tokens of them to really test the hypothesis.)

The most basic measure would be to simply count the number of alternations that occur. However, this must be normalized in some way to make it interpretable. One candidate for normalizing the count of the number of normalizations that occur would be to do so with respect to the size of the set of logically possible alternations for that nominalization. In principle, for any verbal nominalization, the size of the set of possible alternations is equal to the number of possible role-fillers raised to the power of the number of arguments. The number of possible role-fillers is equal to four—arguments can be pre-nominal, post-nominal, NP-external, or absent. So, the size of the set of alternate realizations ranges from a low of four for single-argument predicates (e.g. *occur*) to a high of 4^5^ or 1,024 for a five-argument predicate (e.g. *increase*). In practice, although there are generally no limits on the possibilities for the most “core” arguments (Arg0, Arg1, and often Arg2), the actual possibilities are somewhat lower for other arguments. For example, Arg3 and Arg4 of *increase* (the starting and ending points of the increase) can have at least three realizations, since they may be (1) absent, (2) NP-external, or (3) post-nominal, but it is difficult to get them pre-nominally. Such a nominalization would still, in principle, have 576 possible alternations.

A small number of attested alternation types relative to the possible number of alternation types would be consistent with the predictions of the sublanguage model, while a large number of attested alternation types relative to the possible number of alternation types would seem to falsify it. However, this analysis is untenable for a number of reasons. From a theoretical perspective, it makes an assumption of independence between arguments and of independence between positional variants that is almost certainly not justifiable—for example, it is difficult to get the Arg3 and Arg4 of *increase* (the starting and ending points of the increase) pre-nominally. From an argumentational perspective, it is not convincing to claim that the attestation of all four variants for a single-argument verb constitutes syntactic complexity, nor that the attestation of “only” 21 variants out of a total possible number of 1,024 variants constitutes syntactic simplicity. From a practical perspective, the amount of data that would have to be available to convincingly test the hypothesis is implausibly large—even if no variant occurred more than once, we would need at minimum 1,024 tokens of the noun, which happens to be twice the size of the number of tokens of “increase” and “increases” in the corpus (an unknown number of which are nouns—see footnote 6 above).

One corpus-based approach to characterizing the fit of a textual genre to the sublanguage model is to determine the degree of closure that it exhibits at some level of the grammar. *Closure* is a tendency towards finiteness, e.g. in the size of the lexicon (McEnery and Wilson 2001) [54]. It is evaluated by graphing the number of observations of some phenomenon (e.g. novel word types) as increasingly large amounts of text are examined. Closure manifests as a flattening of the line, while lack of closure manifests as a continual rise in the line. McEnery and Wilson provide an example of such a corpus-based study, looking at closure in the lexicon, in word-type/POS pairs, and shallow parses for three corpora. They demonstrate closure analysis by graphing the increase in the size of the lexicon, in the set of sentential shallow parses, and in the set of word-type/POS pairs against the increase in the total number of words of text examined. This type of analysis could be applied to the data on nominalizations, for which it has a distinct advantage, since it allows all ten nominalizations to be grouped together irrespective of the number of arguments (and hence possible alternations) for the individual nouns. (It is analogous to the case of word-type/POS growth.) However, the amount of text needed for this sort of analysis to be probative is, again, larger than is available in this case.

A compromise position that suggests itself is to consider the ratio of alternations observed to tokens annotated. This is useful, but potentially misleading. Sublanguages typically have a restricted vocabulary relative to other genres, and there is some evidence that this smaller set of lexical items may be coerced into a larger set of syntactic functions. As McEnery and Wilson (2001 [54]:176–180) point out, this leads to a higher type∶token ratio for word-type/POS pairs for the sublanguage than for General English genres, making the sublanguage actually appear more diverse than unrestricted language; however, this ratio fails to account for the difference between the sizes of the lexicon of the sublanguage versus that of the unrestricted genre, and it is only when we graph the growth in count of novel types as increasingly large amounts of text are observed that we see the true closure tendency of the sublanguage.

#### Frequency of the nominalizations

Table 9 lists the nominalizations of the top-10 verbs, ordered by frequency of the corresponding verb in the PennBioIE corpus as a whole, and gives the counts of each nominalization in the corpus as a whole, in the CYP450 section of the corpus, and in the Oncology section of the corpus. (We cannot even estimate the number of tokens of *increase* as a noun in the PennBioIE corpus. As a zero-derived noun, it is tagged incorrectly in revision 0.9 of the corpus (i.e, as a verb) more often than it is tagged correctly.) As in the case of the verbs, the numbers from the GENIA corpus are given for comparison. It is striking how poorly the rank ordering by frequency of the verbs corresponds to the frequency of the nominalizations.

**Table 9:** Counts of the nominalizations in the BioIE and GENIA corpora.

Nominalization	BioIE (both)	BioIE-P450	BioIE-Onc	GENIA
*inhibition*	861	774	87	445
*induction*	342	273	69	826
*increase*	–	–	–	324
*expression*	1,306	300	1,006	3,190
*association*	112	14	98	92
*mediation*	2	1	1	0
*containment*	1	0	1	0
*occurrence*	51	3	48	9
*treatment*	690	477	213	455
*activation*	552	250	302	2,403
**Totals**	3,917	2,092	1,825	7,744

Rows are ordered by frequency of the corresponding verb in the BioIE corpus.

Although a majority are derived by the *-ation* suffix, a surprisingly wide range of derivational morphemes are represented. Only one (*increase*) is zero-related. Two are derived by the non-productive -*ment* suffix— one (*containment*) in its typical use of producing an abstract noun (Bauer & Huddleston [37] p. 1703), and the other (*treatment*) with both the typical abstract noun and the less typical (but possibly more frequent for this lexical item) concrete noun. One (*occurrence*) is non-productive and relatively uncommon. The rest are derived by *-tion* and its variants. This distribution across the word types accords well with the occurrence of derivational suffixes and the word tokens containing them in nominalizations in scientific literature in general—Biber et al. compared the frequencies of the four most common derivational suffixes used to form abstract nouns (-*tion*, *-ity*, *-ism*, *and -ness*) and found that (besides being overwhelmingly more common in academic writing than in conversation, fiction, or news) the *-tion* suffix was the most common suffix, and was more than twice as frequent as the second-most common suffix (*-ity*) and 22 times more frequent than the fourth-most common one (*-ness*) (their Table 4.29, [35] p. 322). They also took productivity into account, and looking specifically at academic prose, differentiated between frequencies of lexicalized and novel nominalizations involving ten nominalizing morphemes, finding that the number of word tokens with *-tion* far outstripped the other nominalizing morphemes in both the lexicalized and the novel categories (their Figure 4.7, [35] p. 323).

#### Incidence of alternations for nominalizations

All nominalizations that occurred more than once evinced multiple alternations, with a range from two (for a nominalization (*mediation*) that only occurred twice) to 24 (for *inhibition*). In the data that follows in this section, we give granular data about the distribution of alternations for the lone single-argument verb and for one of the two-argument verbs, and less granular data for the higher-arity verbs. (Granular data on the distribution of alternations involving Arg0 and Arg1 for *all* verbs is given in Section *Data on alternations involving Arg0 and Arg1 for all predicates*.) In all cases, we exclude from the counts of alternations any nominalization token whose set of arguments included any argument labelled “can't tell.”

Note that compared to the case of the verbal alternations discussed above, quantifying the numbers of alternations observed is much less straightforward for nouns. A number of alternatives are presented earlier in this section; we have tried to combine them here in a way that makes the situation clear.

Table 10 shows the distribution of alternations for *occurrence*. Three alternations were observed. *Occur* is a single–argument predicate, so we could see four different argument realizations; the ratio of possible/observed alternations is 0.75. No tokens of *occur* were labelled “can't-tell.” With three different alternations attested and 51 tokens labelled, the type/token ratio is 0.059. Note that the distribution is extremely skewed—84.3% (43/51) of the tokens had the same pattern, [post-nominal].

**Table 10:** *Occurrence*, the lone single-argument predicate.

	P450	Onc	Both
Pre-nominal	1	4	5
Post-nominal	2	41	43
NP-external	0	3	3
Absent	0	0	0
Total	3	48	51

Tables 11–13 show the distribution of arguments for the two-argument predicator *activation*. (Note that these are not confusion matrices. The value *3* in the *Arg0 Pre/Arg 1 Pre* cell of Table 10 indicates that the nominalization *activation* occurred with both its Arg0 and its Arg1 in the pre-nominal position (e.g. [*Ki-ras_Arg1_*] [*codon 12 point mutational_Arg0_*] *activation*, PMID 10025877, Ko et al. 1998 [55]) three times.) Table 11 gives the data for the entire PennBioIE corpus. Note that the number of alternation types is equal to the number of non-empty cells in the table—in this case, 14. For a two-argument predicator, 16 alternations are possible; the ratio of possible/observed alternations is 0.875 (14/16). 100 tokens were annotated, of which 9 had at least one “can't-tell” argument, and are excluded from the analysis, yielding a type/token ratio of 0.154 (14/91). Table 12 shows the full set of realizations for just the CYP450 section of the corpus, and Table 13 shows it for just the Oncology section. The number of alternations observed does not drop lower than 6. The ratios of possible/attested alternations does not drop lower than 0.375, and the lowest type/token ratio is 0.125 (all three for the Oncology section of the corpus).

**Table 11 pone-0003158-t011:** *Activation*, a two-argument predicator (Arg0 and Arg1).

	Arg0				
		Pre	Post	Ext	Abs
**Arg1**	**Pre**	3	3	1	32
	**Post**	4	6	3	27
	**Ext**	–	1	3	3
	**Abs**	1	1	–	3

Data is combined from both parts of the BioIE corpus. 14/16 possible patterns are attested in 91 tokens (9 can't-tell).

**Table 12 pone-0003158-t012:** Activation, a two-argument predicator (Arg0 and Arg1).

	Arg0				
		Pre	Post	Ext	Abs
**Arg1**	**Pre**	–	3	1	9
	**Post**	1	3	–	14
	**Ext**	–	1	3	3
	**Abs**	1	1	–	3

Data is from the CYP450 section of the corpus. 12/16 possible patterns are attested in 43 tokens (7 can't-tell).

**Table 13:** *Activation*, a two-argument predicator (Arg0 and Arg1).

	Arg0				
		Pre	Post	Ext	Abs
**Arg1**	**Pre**	3	–	–	23
	**Post**	3	3	3	13
	**Ext**	–	–	–	–
	**Abs**	–	–	–	–

Data is from the Oncology section of the corpus. 6/16 patterns are attested in 48 tokens (2 can't-tell).

In Table 14 we show the data on alternations for each of the two-argument verbs. In each case, we give the raw count of alternations, the number of non-“can't-tell” tokens, the ratio of attested/possible alternations, and the type/token ratio.

**Table 14:** Alternations for the four two-argument predicates.

	Alternations	Tokens	X	attested/possible	type/token
*expression*	6	97	4	0.375	0.062
*mediation*	2	2	2	0.124	1.0
*containment*	1	1	0	.063	1.0
*activation*	14	91	9	0.875	0.154

The maximum number possible is 4^2^. Data is given for the full BioIE corpus. The column labelled *tokens* shows the number of tokens for which no argument was labelled “can't tell.” The column labelled *X* shows the number of tokens with at least one argument labelled “can't tell.”

The corresponding data for the three-argument verbs are shown in Table 15. Data for the lone four- and five-argument verbs are combined in Table 16.

**Table 15:** Alternations for the five three-argument predicates.

	Alternations	Tokens	X	attested/possible	type/token
*Inhibition*	24	95	5	0.375	0.253
*Induction*	19	92	8	0.297	0.21
*association.01*	5	8	0	0.078	0.625
*association.02*	10	78	1	0.156	0.128
*treatment.04*	9	58	7	0.141	0.155

The maximum number possible is 4^3^. Data is given for the full BioIE corpus. The column labelled *tokens* shows the number of tokens for which no argument was labelled “can't tell.” The column labelled *X* shows the number of tokens with at least one argument labelled “can't tell.”

**Table 16:** Alternations for the lone 4-argument predicate (*treatment.03*) and the lone 5-argument predicate (*increase*).

	Alternations	Tokens	X	attested/possible	type/token
*treatment.03*	16	29	2	.063	0.552
*Increase*	21	83	17	.021	0.253

The maximum number possible is 4^4^ and 4^5^, respectively. Data is given for the full BioIE corpus. The column labelled *tokens* shows the number of tokens for which no argument was labelled “can't tell.” The column labelled *X* shows the number of tokens with at least one argument labelled “can't tell.”

### Inter-annotator agreement

#### Inter-annotator agreement for verbal alternations

With about fifteen minutes' worth of training, inter-annotator agreement for the verb data was 78%. The majority of the disagreements were mismatches in transitivity judgements and were related to the single word-form *expressed*. Specifically, many tokens that we tagged as *PT* were tagged as PI by the second annotator, and many tokens that we tagged as *pt* were tagged as *pi* by the second annotator. We generally agreed on the active/passive contrast and on the verbal/adjectival contrast.

#### Inter-annotator agreement for nominalizations

Inter-annotator agreement data for nominalizations is given in Table 17. The annotation task included selecting and labelling the nominalization itself; although this process was manual, and choices had to be made about span selection and about sense selection for the nominalizations *association* and *treatment*, there were no sense selections to be made for the two verbs for which we calculated IAA data, and the span selection guidelines for the nominalizations themselves were quite straightforward, so it is not surprising that the IAA for predicator selection was trivially quite high at 100%. IAA for the arguments is more interesting.

**Table 17:** Inter-annotator agreement for the two most difficult nominalizations.

	IAA	TP	FP	FN
Both predicates	100%	28	0	0
All arguments for both predicators	87.5%	49	7	7
Positionally defined types for both predicators	95.8%	34	3	0
Other types for both predicators	68.4%	13	5	7
Arg0 for both	74.1%	20	7	7
Arg1 for both	96.4%	27	1	1

With about two hours' training for the second annotator, overall IAA across all arguments for both predicates was 87.5%. (For comparison: Meyers et al. (2004a) [32] reported “inter-annotator consistency scores ranging from 82% to 90%” during the training phase (p. 803). Palmer et al. (2005) [2] reported kappa of 0.91 for a more constrained task than the one that we describe here.) IAA varied considerably between the two positionally-defined types (i.e., pre- and post-nominal arguments) and the others: across both predicators, IAA for the positionally defined argument types was 95.8%, falling to 68.4% for the others. Arg0s and Arg1s also differed markedly—IAA for Arg0 across both predicators was only 74.1%, while IAA for Arg1 was 96.4%. These findings are almost certainly related: Arg0s were absent much more often than Arg1s, and as the confusion matrices will show, absent arguments and NP-external arguments were frequently associated with disagreements between annotators. This accords generally with the findings of Meyers et al. (2004b) [33], who reported inter-annotator agreement rates of around 85% and below and noted that their primary sources of disagreements were “SUPPORT verbs and the shared arguments that go with them [NP-external, in this paper]…role assignment to prenominals…[and] errors” (p. 28).

Tables 18 through [Table pone-0003158-t019] [Table pone-0003158-t020] [Table pone-0003158-t021] [Table pone-0003158-t022] [Table pone-0003158-t023] 24 give the confusion matrices for various views on the data. Columns are the primary annotator's judgements, while rows are the second annotator's judgements. Fractions are the count for that cell divided by the number of slots labelled (which varies from table to table and is identified in the table caption). Following Palmer et al. (2005) [2], we include matches in the confusion table, and so the mismatches are percentages of the total judgements, not percentages of the errors. Table 18 gives the overall picture. The positionally defined categories (pre- and post-nominal) contributed the smallest number of disagreements overall. The largest overall contributor of disagreements was confusion of absent arguments with NP-external arguments.

**Table 18:** Confusion matrix for both nominalizations and both arguments.

	Pre	Post	Ext	Abs	X
**Pre**	.321		.018		
**Post**	.018	.286		.018	
**Ext**			.089	.089	
**Abs.**				.143	
**X**				.018	

Confusion between NP-external and absent arguments was the largest source of disagreements. Fractions are the count for the cell divided by the number of slots (56). They sum to 1.

**Table 19 pone-0003158-t019:** Confusion matrix for Arg0 of both nominalizations.The denominator is 28.

	Pre	Post	Ext	Abs.	X
**Pre**	.036		.036		
**Post**		.25		.036	
**Ext**			.143	.179	
**Abs.**				.286	
**X**				.036	

**Table 20 pone-0003158-t020:** Confusion matrix for Arg1 of both nominalizations.

	Pre	Post	Ext	Abs.	X
**Pre**	.61				
**Post**	.036	.32			
**Ext**			.036		
**Abs.**					
**X**					

The denominator is 28.

**Table 21 pone-0003158-t021:** Confusion matrix for Arg0 of *activation*.

	Pre	Post	Ext	Abs	X
**Pre**	.071		.071		
**Post**		.286			
**Ext**			.143	.143	
**Abs.**				.286	
**X**					

The denominator is 14.

**Table 22 pone-0003158-t022:** Confusion matrix for Arg1 of *activation*.

	Pre	Post	Ext	Abs	X
**Pre**	.643				
**Post**		.286			
**Ext**			.071		
**Abs.**					
**X**					

The denominator is 14.

**Table 23 pone-0003158-t023:** Confusion matrix for Arg0 of *expression*.

	Pre	Post	Ext	Abs.	X
**Pre**					
**Post**		.214		.071	
**Ext**			.143	.214	
**Abs.**				.286	
**X**				.071	

**Table 24:** Confusion matrix for Arg1 of *expression*.

	Pre	Post	Ext	Abs.	X
**Pre**	.61				
**Post**	.036	.32			
**Ext**			.036		
**Abs.**					
**X**					

Tables 19 and 20 demonstrate that overall, Arg0 is the major source of disagreements.

Tables 21–22 and Tables 23–24 show that this is true for the individual nominalizations, as well: IAA is lower for Arg0 than for Arg1 for both nominalizations, and not just in the aggregate.

### Semantic roles and syntactic position

Palmer et al. examined associations between semantic roles and syntactic roles (e.g. subject, object, and SBAR), finding “evidence for the notion of a thematic hierarchy in which the highest-ranking role present in a sentence is given…subjecthood” (2005 [2]:90–91). This analysis cannot be carried out for the nominalization data, since the notion of syntactic role for nominalizations is not well-defined (*pace* Roeper and van Hout). However, associations between semantic roles and syntactic *positions* relative to the nominalization are testable with this data. Following Palmer et al.'s analysis, Tables 25 and 26 show the most frequent syntactic positions for each semantic role, and the most frequent semantic role for each syntactic position.

**Table 25:** The most frequent syntactic positions for each semantic role (cf. Palmer et al.'s Table 7, 2005:91).

Semantic role	Total	Most common syntactic positions
**Arg0**	570	Absent (378), NP-external (82), Post-nominal (64), Pre-nominal (46)
**Arg1**	612	Post-nominal (341), Pre-nominal (124), Absent (79), NP-external (68)

See Tables 43 and 44 for the raw data.

**Table 26:** The most frequent semantic roles for each syntactic position (c.f. Palmer et al.'s Table 6, 2005:90).

Position	Total		
**Pre-nominal**	Arg1 (124)	Arg0 (51)	175
**Post-nominal**	Arg1 (341)	Arg0 (107)	448
**NP-external**	Arg0 (85)	Arg1 (68)	153
**Absent**	Arg0 (378)	Arg1 (79)	461

Only Args 0 and 1 are indicated. *Association.02,03* are omitted. See Tables 43 and 44 for the raw data.

Note that the *post-nominal* position blurs the distinction between complements and adjuncts, which may well be relevant here, as well as potential diathesis alternations specific to ditransitives, and possibly other interesting phenomena, as well.

If contrasting these numbers with Palmer et al.'s findings for verbs, note also that the numbers of semantic and syntactic roles are both much larger in PropBank.

Examining the most frequent syntactic positions for each semantic role, the most striking finding is that for Arg0, the most frequent syntactic position is complete absence. This finding accords with the general observation that nominalization is similar to passivization in that it allows for the omission of agents. (It also helps to explain the most frequent semantic roles that we observe for each syntactic position—we return to this point momentarily.) The next most frequent syntactic position for Arg0 is NP-external. This implies a real challenge for semantic role labelling: the inter-annotator agreement data indicates that even for humans, this distinction was the most difficult to make.

For Arg1, two points are evident. The first is that the pre-nominal position, although not the *most* common, is nonetheless quite commonly observed in this data. The second is that about a quarter of the nominalizations in the data have an Arg1 that is either absent or NP-external; on most analyses, these are intransitives (specifically, unergatives), demonstrating that this alternation is attested in nouns, as well as in verbs, in this domain.

These findings emphasize the importance of dealing with pre-nominal arguments in biomedical information extraction systems: 20% (124/612) of all Arg1s and 8% (46/570) of all Arg0s in this data are pre-nominal. Similarly, we see the importance of the ability to recognize when arguments are entirely absent: 66% (378/570) of all Arg0s and 13% (79/612) of all Arg1s in this data are absent. This finding argues strongly against the NomBank policy of not annotating argumentless nominalizations at all. (Higher-arity predicates may lack overt Arg0 and Arg1, but still have other arguments present—we return to this point below.)

Finally, the data on the most frequent syntactic positions for each semantic role are a reflection of the data on the most frequent semantic role for each syntactic position. Table 26 shows that for the syntactic positions within the noun phrase, Arg1s outnumber Arg0s by at least a ratio of 3∶1. This is not surprising given the data in Table 25, as well as in the fourth row of Table 26: overall, non-absent Arg1s simply outnumber non-absent Arg0s in the data, so it is not surprising that they would tend to outnumber them in any given (non-absent) syntactic position. (A single one of the ten predicates (*occur*) considered in this work has an Arg1, but no Arg0, and this fact makes a small contribution to the ratio of Arg0s and Arg1s potentially present; however, only 51 tokens of this nominalization were present in the data, so the contribution is not large.)

### Nominalizations with no overt arguments

The granular data presented in this paper allows us to evaluate the consequence of the NomBank decision not to annotate nominalizations that have no overt arguments. (In this work, we annotated all nominalizations, whether or not there were arguments present—see Section 1.4, *Contrasts between this work and NomBank*.)

Table 27 gives the data on the incidence of argumentless nominalizations: about 12% of the verbal nominalizations in this data have no Arg0 or Arg1. (Note that since we did not distinguish between core and adjunctive arguments, treating all arguments as core arguments, while NomBank preserves the core/adjunct distinction, Arg0/Arg1 are probably a better estimate of the incidence of argumentless nouns in projects like NomBank than are the “no arguments at all” row in Table 27.) For some predicates, Arg0/Arg1-less tokens were near-majorities or even near-total—22/29 tokens of *treatment.03* had no Arg0 or Arg1, while 26/58 tokens of *treatment.04* had no Arg0 or Arg1. Clearly, NomBank's decision not to annotate argumentless nominalizations results in omission of a non-trivial amount of data from the analysis, at least in the genre under examination in this paper. It also means sacrificing otherwise very clear generalizations about the behavior of specific predicates. Other implications of this choice (and a potential solution) are discussed below in Section *Implications for annotation efforts*.

**Table 27:** Argumentless nominalizations.

No arguments at all	20
No Arg0 or Arg1	71

### Data on alternations involving Arg0 and Arg1 for all predicates

Tables 28 through [Table pone-0003158-t029] [Table pone-0003158-t030] [Table pone-0003158-t031] [Table pone-0003158-t032] [Table pone-0003158-t033] [Table pone-0003158-t034] [Table pone-0003158-t035] [Table pone-0003158-t036] [Table pone-0003158-t037] [Table pone-0003158-t038] [Table pone-0003158-t039] [Table pone-0003158-t040] 41 give granular data on each nominalization. Contra the tables in the body of the text, the number of alternations in this appendix is *not* necessarily equal to the number of non-empty cells, since the tables only show patterns involving Args 1 and 2—for predicates with greater than two arguments, it may be larger. See the earlier tables in the body of the paper for the number of alternations attested for such verbs.

**Table 28:** *Occurrence*, a 1-argument predicator (Arg1).

	P450	Onc	Both
**Pre-nominal**	1	4	5
**Post-nominal**	2	41	43
**NP-external**	0	3	3
**Absent**	0	0	

Data is combined from both parts of the BioIE corpus. 3/4 possible patterns are attested in 51 tokens (0 can't-tell).

**Table 29 pone-0003158-t029:** *Activation*, a two-argument predicator (Arg0 and Arg1).

	Arg0				
		Pre	Post	Ext	Abs
**Arg1**	**Pre**	3	3	1	32
	**Post**	4	6	3	27
	**Ext**	–	1	3	3
	**Abs**	1	1	–	3

Data is combined from both parts of the BioIE corpus. 14/16 possible patterns are attested in 91 tokens (9 can't-tell).

**Table 30 pone-0003158-t030:** *Activation*, a two-argument predicator (Arg0 and Arg1).

	Arg0				
		Pre	Post	Ext	Abs
**Arg1**	**Pre**	–	3	1	9
	**Post**	1	3	–	14
	**Ext**	–	1	3	3
	**Abs**	1	1	–	3

Data is from the CYP450 section of the corpus. 12/16 possible patterns are attested in 43 tokens (7 can't-tell).

**Table 31 pone-0003158-t031:** *Activation*, a two-argument predicator (Arg0 and Arg1).

	Arg0				
		Pre	Post	Ext	Abs
**Arg1**	**Pre**	3	–	–	23
	**Post**	3	3	3	13
	**Ext**	–	–	–	–
	**Abs**	–	–	–	–

Data is from the Oncology section of the corpus. 6/16 patterns are attested in 48 tokens (2 can't-tell).

**Table 32 pone-0003158-t032:** *Inhibition*, a 3-argument predicator (Arg0, Arg1, and Arg2; only Args 0 and 1 are shown).

	Arg0				
		Pre	Post	Ext	Abs
**Arg1**	**Pre**	–	2	8	4
	**Post**	1	15	16	26
	**Ext**	1	3	5	1
	**Abs**	3	2	2	6

Data is combined from both parts of the BioIE corpus. 24/64 possible patterns are attested in 95 tokens (5 can't-tell).

**Table 33 pone-0003158-t033:** *Induction*, a 3-argument predicator (Arg0, Arg1, and Arg2; only Args 0 and 1 are shown).

	Arg0				
		Pre	Post	Ext	Abs
**Arg1**	**Pre**	1	3	–	8
	**Post**	11	12	3	33
	**Ext**	3	2	2	3
	**Abs**	2	1	–	8

Data is combined from both parts of the BioIE corpus. 19/64 possible patterns are attested in 92 tokens (8 can't-tell). For comparability with other tables in the paper, tokens where any arg is X are omitted from this table, but there are 3 additional tokens where the X is in Arg2 that could be added to this table: 1 Arg0-Ext/Arg1-Ext, and 1 Arg0-Ext/Arg1-Abs.

**Table 34 pone-0003158-t034:** *Increase*, a 5-argument predicator (Arg0, Arg1, Arg2, Arg3, and Arg4; only Args 0 and 1 are shown).

	Arg0				
		Pre	Post	Ext	Abs
**Arg1**	**Pre**	–	–	–	7
	**Post**	6	10	27	30
	**Ext**	–	1	2	3
	**Abs**	–	–	–	2

Data is combined from both parts of the BioIE corpus. 21/1,024 (4^5^) possible patterns are attested in 83 tokens (17 can't-tell).

**Table 35 pone-0003158-t035:** *Expression*, a 2-argument predicator (Arg0 and Arg1).

	Arg0				
		Pre	Post	Ext	Abs
**Arg1**	**Pre**	–	1	1	42
	**Post**	–	–	3	44
	**Ext**	–	–	–	6
	**Abs**	–	–	–	–

Data is combined from both parts of the BioIE corpus. 6/16 possible patterns are attested in 97 tokens (4 can't-tell).

**Table 36 pone-0003158-t036:** *Association.01*, a 3-argument predicator (Arg0, Arg1, and Arg2; only Args 0 and 1 are shown).

	Arg0				
		Pre	Post	Ext	Abs
**Arg1**	**Pre**	–	–	1	–
	**Post**	–	–	2	–
	**Ext**	–	–	3	2
	**Abs**	–	–	–	–

Data is combined from both parts of the BioIE corpus. 5/64 possible patterns are attested in 8 tokens (0 can't-tell).

**Table 37 pone-0003158-t037:** *Association.02* (reciprocal); only Args 0 and 1 for non-plural associands are shown.

	Arg0				
		Pre	Post	Ext	Abs
**Arg1**	**Pre**	–	–	–	1
	**Post**	8	39	13	4
	**Ext**	1	–	8	–
	**Abs**	–	–	–	4^8^

Data is combined from both parts of the BioIE corpus. 10 patterns are attested in 78 tokens (1 can't-tell); the number of possible patterns is greater than 64, but its exact value depends on how reciprocal tokens are handled.

**Table 38 pone-0003158-t038:** *Mediation*, a 2-argument predicator.

	Arg0				
		Pre	Post	Ext	Abs
**Arg1**	**Pre**	–	–	–	–
	**Post**	1	1	–	–
	**Ext**	–	–	–	–
	**Abs**	–	–	–	–

Data is combined from both parts of the BioIE corpus. 2/16 possible patterns are attested in 2 tokens (2 can't-tell).

**Table 39 pone-0003158-t039:** *Containment*, a 2-argument predicator.

	Arg0				
		Pre	Post	Ext	Abs
**Arg1**	**Pre**	–	–	–	1
	**Post**	–	–	–	–
	**Ext**	–	–	–	–
	**Abs**	–	–	–	–

Data is combined from both parts of the BioIE corpus. 1/16 possible patterns are attested in 1 token (0 can't-tell).

**Table 40 pone-0003158-t040:** *Treatment.03* (medical), a 4-argument predicator (only Args 0 and 1 are shown).

	Arg0				
		Pre	Post	Ext	Abs
**Arg1**	**Pre**	–	–	–	1
	**Post**	–	–	–	3
	**Ext**	–	–	–	3
	**Abs**	–	–	–	22

Data is combined from both parts of the BioIE corpus. 16/256 (4^4^) possible patterns are attested in 29 tokens (2 can't-tell).

**Table 41:** *Treatment.04* (affect a change in something by applying a substance), a 3-argument predicator (only Args 0 and 1 are shown).

	Arg0				
		Pre	Post	Ext	Abs
**Arg1**	**Pre**	–	–	–	–
	**Post**	–	–	–	14
	**Ext**	–	–	–	18
	**Abs**	–	–	–	26

Data is combined from both parts of the BioIE corpus. 9/64 possible patterns are attested in 58 tokens (7 can't-tell).

### Data on role/position and position/role associations

Table 42 gives the counts of each syntactic position for Arg0 for each individual predicate. Table 43 gives the same data for Arg1. In both tables, we included the data for *association.01* but excluded all data for *association.02* and .*03*.

**Table 42:** Most frequent syntactic positions for Arg0 (*association.02,.03 (reciprocal*, and .*03* omitted).

	Total	Arg0 absent	Arg0 external	Arg0 post-nom	Arg0 pre-nom
Occurrence		–	–	–	–
Activation		65	7	11	8
Inhibition		37	31	22	5
Induction		52	5	18	17
Increase		42	29	11	6
Expression		92	4	1	0
Association.01		2	6	0	0
Mediation		0	–	1	1
Containment		1	0	0	0
Treatment.03		29	0	0	0
Treatment.04		58	0	0	0
Total		378	82	64	46

Note that occurrence is a single-argument predicate and has no Arg0.

**Table 44:** Most frequent syntactic positions for Arg1.

	Total	Arg1 post-nom	Arg1 pre-nom	Arg1 absent	Arg1 external
Occurrence		43	5	0	3
Activation		40	39	5	7
Inhibition		58	14	13	10
Induction		59	12	11	10
Increase		73	7	2	6
Expression		47	44	0	6
Association.01		2	1	0	5
Mediation		2	0	0	0
Containment		0	1	0	0
Treatment.03		3	1	22	3
Treatment.04		14	0	26	18
Total		341	124	83	68

## Discussion

### Implications for annotation efforts

In the Introduction, we reviewed the implications of our findings for biomedical language processing. Here, we discuss the implications of our work for annotation efforts. A major focus of this paper is alternations involving nominalizations. Our annotation guidelines differed in a number of ways from that of NomBank, the only large-scale effort to annotate the argument structures of nominalizations to date (see Section *Contrasts between this work and NomBank annotations*).

These differences allow us to evaluate the consequences of one of NomBank's design decisions: specifically, the project's decision not to annotate nominalizations that have no overt arguments. The data presented here shows that a non-trivial proportion of the nominalization tokens in our data have no arguments at all, and that some nominalizations lack arguments more often than not. If applied to this data, NomBank's decision not to annotate argumentless nominalizations would come with a number of costs.

Some of these would hurt the researcher theoretically: we would lose a non-trivial amount of data for our analysis, and the frequent omission of arguments is clearly a salient characteristic of the behavior of some lexical items. If we are concerned with understanding alternations for the light that they shed on the relationship between syntax and semantics, we would be at a minimum eliminating a number of evidently intransitive nouns from consideration completely.

Other costs would have practical consequences: we are less likely to improve the comparatively low performance of the current nominalization semantic role labelling systems if we cannot learn to recognize when arguments are absent, and we are less likely to recognize when they are absent if we deliberately exclude argumentless nouns from our data. The high incidence of the difficult-to-distinguish *Absent* and *NP-external* categories highlights the importance of being able to make this distinction. The data presented here suggests that, *pace* Meyers and his coworkers, argumentless nouns should not be excluded from annotation efforts. It is possible that automatic methods could be effective in ameliorating the effects of that decision for the specific case of the NomBank effort at relatively low cost.

### Related work

Hirschman and Sager (1982) [56] discuss alternations in the semantic classes of verb arguments, e.g. the alternation between lab tests and body parts as the subject of the verb **show** in medical texts (*X-rays of spine show extreme arthritic change* versus *The dorsal spine shows moderately severe degenerative changes…*, p. 34), but do not focus on syntactic alternations.

The only previous quantitative data on alternations that we are aware of appears in Biber et al. (op cit). They give data on the relative incidence of valence types across broad semantic domains such as *activity*, *communication*, *occurrence*, and *existence*, finding that verbs with at least one transitive pattern are the most common, 36% of common verbs occurring transitively only, 47% occurring both transitively and intransitively. Just 10 verbs in total occurred solely intransitively (p. 382). For the verbs that can participate in transitivity alternations, they found that although there are clear verb-specific differences, transitive uses tended to be more common. The PennBioIE data accords with both of these General English tendencies.

We are not aware of any quantitative data on alternations involving nominalizations. Herbst et al. (2004) [57] is a corpus-based description of the valency patterns of 511 verbs, 274 nouns, and 544 adjectives (p. XL). A small fraction of the valency patterns listed in the book contain very non-granular indications of their relative frequencies: “Patterns for which only a few instances could be found in the corpus are labelled *rare*” (p. XL). A subset of verbal patterns, and a different subset of nominal and adjectival patterns, are marked as “>30%” if that pattern makes up at least 30% of all occurrences of the word in the corpus, as “very frequent” if the pattern “is significantly more frequent than other…patterns,” or “frequent” if the pattern is “relatively frequent.” No information is available on the number of patterns in the book that have even this very coarse sort of quantitative data, but impressionistically, it appears to be only a fraction of the patterns presented in the book.

### Alternations and semantic representations

The low representation of Change Of State verbs in the top-ten list is surprising. Biologists conceive of many molecular events as “state-changing,” but only the single predicate *increase* clearly has such semantics. Table 44 gives the Levin class assignments of the top-10 verbs. Levin classes that seem to fit the domain-specific meanings attested in the corpus are bolded. The predicate *increase* is the single clear example of a Change Of State verb. Only seven of the ten verbs appear in Levin (1993) [1] at all, and two of those seven verbs are not adequately represented in Levin (1993) [1] with respect to their biomedical semantics. In this domain, *express* behaves more like a *Create* verb (Levin class 26.4) than like either of the classes to which it is assigned. (This sense is not found in WordNet 2.1, either.) *Treat* has at least two senses in this domain, neither of which is represented in Levin (1993) [1], one representing WordNet 2.1's process, treat synset, and the other corresponding to the treat, care **for** synset. Filling these gaps in the representations of these verbs seems worthwhile.

**Table 45:** Levin classes of the most common verbs.

Lemma	Class	
inhibit	—	—
induce	—	—
increase	45.4	Verbs of Change of State: Other alternating verbs of change of state
	**45.6**	**Verbs of Change of State: Verbs of calibratable change of state**
express	11.1	Verbs of Sending and Carrying: *Send* verbs
	48.1.2	Reflexive verbs of appearance
associate	**22.2**	**Verbs of Combining and Attaching: ** ***Amalgamate*** ** verbs**
mediate	—	—
contain	8.2	Verbs Requiring Special Diatheses: Obligatorily reflexive object
	**47.8**	**Verbs of Existence: Verbs of contiguous location**
	54.3	Measure Verbs: *Fit* verbs
occur	**48.3**	**Verbs of Appearance, Disappearance, and Occurrence: Verbs of occurrence**
treat	8.5	Verbs Requiring Special Diatheses: Obligatory adverb
	29.2	Verbs with Predicative Complements: *Characterize* verbs

Verbs are ordered by frequency. Dashes indicate that the verb does not appear in Levin (1993). Bolding indicates that the Levin class seems to fit the semantics of the verb as used in the CYP450 section of the BioIE corpus.

In light of the high incidence of alternations in the corpus, it is notable that the majority of the top-ten verbs are result verbs, rather than manner verbs. Rappaport-Hovav and Levin's (1998) [58] work on semantic representation of verb meaning predicts that result verbs participate in a smaller number of alternation types than manner verbs; apparently this does not correlate with a low number of alternation “tokens.”

Although our data suggest that alternations in this genre are frequent, this finding does not contradict either the sublanguage hypothesis itself, or Friedman et al.'s claim that scientific abstracts in this domain fit the sublanguage model. The sublanguage model predicts that the range of syntactic and semantic phenomena in a sublanguage will be *limited*, but it does not necessarily claim that they will be *simple*. Indeed, the history of sublanguage research includes a number of phenomena that are syntactically and semantically quite complex, e.g. Dunham (1986) [59] on noun phrases, Finin (1986) [60] on compound nouns, and ellipsis and anaphora in recipes and technical manuals (Kittredge 1982 [61], Palmer et al. 1986 [62]). Indeed, some of the phenomena that we identify as troublesome for biomedical IE systems are quite repetitive and amenable to relatively simple interpretation rules. For example, of the 59 tokens of the transitive present participial adjective alternation, 20 of those tokens fit the pattern *NP1-containing NP2*, where the semantic relation is that NP2 contains NP1. Of the 184 tokens of adjectival passive alternations, 13 (7%) are the single type *cDNA-expressed*, and 12 (6.5%) are some variant surface form of the single underlying concept *calcium-activated potassium channel*. Similar phenomena are observable in the nominalizations—recall the skewedness of the distributions of alternations for the two nominalizations for which we saw granular data (Table 9 for *occurrence* and Tables 10–12 for *activation*). For example, of the 101 tokens of *expression* that we annotated, 44.6% (45/101) had the Arg1 in pre-nominal position, while 48.5% (49/101) had it in the post-nominal position. Of the 49 post-nominal Arg1s for *expression*, in a full 39 cases the Arg1 was in an immediately adjacent prepositional phrase headed by the preposition *of*; in the remaining 10 cases, the PP was still an *of* -phrase (although either an intervening head noun or another conjoined nominalization intervened). So, despite the semantic and syntactic complexity, the predictions of the sublanguage model hold.

### Future directions

There are a number of future directions for this work.

A methodological one would be to extend the annotation work beyond verbal nominalizations to include argument nominalizations (e.g. agentives). Examining alternations involving additional types of nominalizations would round out our picture of syntactic and semantic variability in this domain.

There are also additional theoretical directions in which this work could be taken. The data in this paper answered the basic question that we set out to ask: do alternations occur in biomedical text? With the nominalization data in hand, a number of deeper questions can now be addressed. Some of these are big questions. Having seen that alternations occur, it would be interesting to ask if the actual alternations attested correlate with the semantics of the verbs, as Levin would lead us to expect—granular analyses like the data that we present for *activation* suggest that alternations involving verbal nominalizations are exactly the “intricate and extensive patterns of syntactic behavior” that Levin (1999 [1]:16) suggests will lead us to an understanding of relationships between semantics and syntax. Other questions are much more specific. For example, Roeper and van Hout's 2006 paper [44] makes a strong claim: “Our theory depends upon a claim that extends to all affixation: affixes determine argument structure.” This counter-intuitive claim could be investigated with data like that which is presented in this paper, for a larger number of verbs and derivational morphemes.
